# Role of the Stress- and Inflammation-Induced Cytokine GDF-15 in Cardiovascular Diseases: From Basic Research to Clinical Relevance

**DOI:** 10.31083/j.rcm2403081

**Published:** 2023-03-06

**Authors:** Anja Schwarz, Ralf Kinscherf, Gabriel A. Bonaterra

**Affiliations:** ^1^Department of Medical Cell Biology, Institute for Anatomy and Cell Biology, Philipps-University of Marburg, 35037 Marburg, Germany

**Keywords:** GDF-15, inflammation, stress, coronary artery disease

## Abstract

Stress- and inflammation-induced growth differentiation factor-15 (GDF-15) is proposed as a biomarker for mortality and disease progression in patients with atherosclerosis and/or cardiovascular disease (CVD). The development of atherosclerotic lesions depends, among other factors, on inflammatory processes, oxidative stress, and impaired lipid homeostasis. As a consequence, activation and dysfunction of endothelial cells, release of chemokines, growth factors and lipid mediators occur. GDF-15 is suggested as an acute-phase modifier of transforming growth factor (TGF)-ßRII-dependent pro-inflammatory responses leading to rupture of atherosclerotic plaques, although the exact biological function is poorly understood to date. GDF-15 is upregulated in many disease processes, and its effects may be highly context-dependent. To date, it is unclear whether the upregulation of GDF-15 leads to disease progression or provides protection against disease. Concerning CVD, cardiomyocytes are already known to produce and release GDF-15 in response to angiotensin II stimulation, ischemia, and mechanical stretch. Cardiomyocytes, macrophages, vascular smooth muscle cells, endothelial cells, and adipocytes also release GDF-15 in response to oxidative as well as metabolic stress or stimulation with pro-inflammatory cytokines. Given the critically discussed pathophysiological and cellular functions and the important clinical significance of GDF-15 as a biomarker in CVD, we have summarized here the basic research findings on different cell types. In the context of cellular stress and inflammation, we further elucidated the signaling pathway of GDF-15 in coronary artery disease (CAD), the most common CVD in developing and industrial nations.

## 1. Introduction

Growth differentiation factor-15 (GDF-15), which is identical to macrophage inhibitory cytokine-1 (MIC-1), prostate-derived factor (PDF), nonsteroidal anti-inflammatory drug (NSAID)-activated gene-1 (NAG-1), placental bone morphogenetic protein (PLAB), and placental transforming growth factor (PTGF) [1, 2, 3, 4, 5, 6], is a divergent member of the transforming growth factor-β (TGF-β) superfamily [7]. The unprocessed, translated pre-pro-GDF-15 form consists of 308 amino acids, yielding a 40 kDa pro-peptide monomer that is finally processed into a mature 30 kDa secreted homodimer peptide linked by disulfide bonds [1]. GDF-15 is soluble and circulates in the bloodstream, where its concentration can be measured. Under physiological conditions human sera contain 0.15–1.15 ng/mL GDF-15 [8, 9, 10, 11]. Additionally, GDF-15 is also widely distributed in adult tissues [12]: Specifically, in the cardiovascular system, GDF-15 is expressed in various cell types, e.g., cardiomyocytes, macrophages, endothelial cells (ECs), vascular smooth muscle cells (VSMCs), but also in adipose tissue [2, 13, 14, 15].

**Fig. 1.**
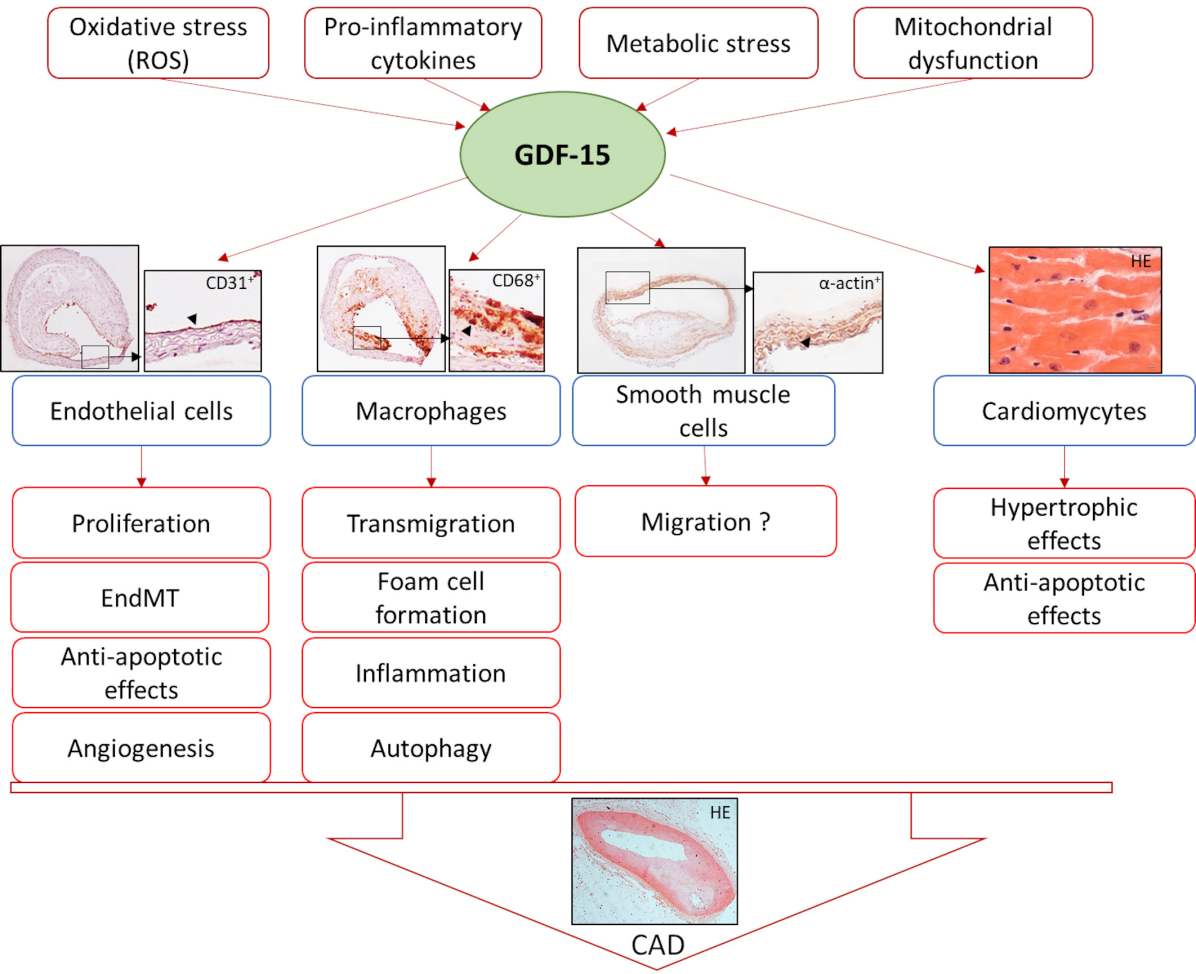
**Induction of GDF-15 and its effects on different cell types leading to coronary artery disease (CAD)**. HE, Hematoxylin-eosin stain.

Many clinical trials revealed elevated plasma/serum GDF-15 levels in various diseases, thus, indicating that GDF-15 may be considered as a biomarker. These pathophysiological conditions and diseases associated with increased plasma/serum GDF-15 levels include endothelial activation and vascular inflammation, which determine the development and progression of atherosclerosis, cardiovascular disease (CVD) and/or cardiometabolic diseases [16, 17, 18], heart failure [19, 20], lipodystrophy [15, 21], or even cancer [10, 22, 23, 24, 25, 26]. With respect to CVD, macrophages, VSMCs, ECs, adipocytes, and cardiomyocytes produce and release GDF-15 in high concentrations in response to mitochondrial dysfunction, oxidative stress, metabolic stress, and/or through stimulation by pro-inflammatory cytokines [1, 13, 15, 27, 28] (Fig. 1).

Previous studies have shown, that the physiological effect of GDF-15 is highly context-dependent and can vary significantly with the stage of disease [29]. Therefore, we would like to use this review to summarize the actual existing research data and focus on the effect of GDF-15 in different cell types with special reference to cellular stress and inflammation to better understand the signaling pathways of GDF-15 in coronary artery disease (CAD).

## 2. Implications of GDF-15 in CAD—Clinical Data/Trials

CAD, also named coronary heart disease (CHD), ischemic heart disease (IHD), or myocardial ischemia is a chronic heart disease caused by atherosclerotic plaques in the coronary arteries leading to more or less coronary stenosis. In this context, several studies have shown that GDF-15 is useful as a consistent biomarker of mortality and CV events in patients with acute coronary syndrome (ACS) [30, 31, 32, 33, 34], acute Heart Failure [35] or stable CAD [34, 36, 37, 38, 39] (Table 1, Ref. [30, 32, 33, 34, 37, 38, 39, 40, 41, 42].

**Table 1.** **GDF-15 in terms of cardio vascular events in representative studies**.

Study	Population	Median GDF-15 concentration at baseline (ng/L)	GDF-15 assays	Follow up (Median)	Reference
Gusto-4	2081 patients; NSTE-ACS	1434 (1035–2078) (validation cohorte)	IRMA	1 year	[30]
		1499 (1151 to 2203) (derivation cohort)			
Assent-2 and assent-plus	741 patients; STEMI	1635 (1164–2309)	IRMA	1 year	[33]
Atherogene	1352 patients with SAP, 877 patients with ACS	SAP: 1128 (850–1553) ACS: 1244 (962 to 1785)	immunoradiometric assay (IRMA)	3.6 years	[34]
Leicester royal infirmary infarct registry	1142 patients; NSTEMI or STEMI	1470 (240–31,860)	ELISA (antibodies from R&D)	1.4 years	[32]
Prove it-timi-22	3501 patients; NSTE-ACS or STEMI	1362 (1032–1844)	IRMA	2 years	[41]
Heart and soul	984 patients; stable CHD	2166 (1589–3057)	Luminex Sandwich Assay (Alere Diagnostics, San Diego, CA)	8.9 years	[39]
Iabp-shock-2	190 patients NSTEMI or STEMI and cardiogenic shock undergoing primary PCI	7662	Quantikine ELISA (R&D)	30 days	[42]
Karola	1029 patients; stable CHD, History of MI or CABG	1232 (916–1674)	ElectroChemi-Luminescence Immunoassays (Fa. Roche)	10 years	[38]
Plato	16,876 patients NSTE-ACS or STEMI	1550 (1145–2219)	ElectroChemi-Luminescence Immunoassays (Fa. Roche)	1 year	[40]
Stability	14,577 patients; stable CHD	1253 (915–1827)	ElectroChemi-Luminescence Immunoassays (Fa. Roche)	3.7 years	[37]

The GUSTO-4 (Global Utilization of Strategies to Open Occluded Arteries-4) trial demonstrated a strong association between GDF-15 concentration in the blood of patients at hospital admission and all-cause mortality in non-ST-segment-elevation ACS (NSTE-ACS) [30] (Table 1). In the samples of 2081 patients with NSTE-ACS, increasing levels of GDF-15 at admission were positively associated with age, female sex, hypertension, and diabetes [30]. GDF-15 levels were also associated with previous manifestations of heart disease, current angiotensin-converting enzyme inhibitor therapy, and markers of ongoing ischemia and necrosis, myocardial dysfunction, and inflammation [30]. In addition to independent risk indicators such as, age, N-terminal pro-brain natriuretic peptide (NT-proBNP), and myocardial infarction, GDF-15 was the most important predictor of death in this study [30]. By determining 1-year cumulative mortality rates, GDF-15 was one of the best predictors provided prognostic information more than other clinical biomarkers (cardiac troponin-T [cTnT], NT-proBNP, hs- C-reactive protein [CRP], and creatinine clearance) in the comparison [30]. In patient groups with NSTE-ACS or ST-elevation myocardial infarct (STEMI), the independent association of GDF-15 with mortality was reconfirmed [32, 33]. In patients with NSTE-ACS or STEMI the prognostic value of GDF-15 was reassessed, in the Platelet Inhibition and Patient Outcomes Trial (PLATO) [40] (Table 1). Because of the large number of patients, the PLATO biomarker study examined the association between GDF-15 and specific outcome events during follow-up. After adjusting for clinical predictors and biomarkers (hs-cTnT, NTproBNP, hs-CRP, and cystatin C), the study showed that elevated GDF-15 levels were associated with an increased risk of, CV mortality, myocardial infarction, and stroke [40].

The AtheroGene registry enrolled patients with stable angina pectoris (SAP) or ACS who underwent coronary angiography and had stenosis of >30% in the main coronary arteries [34] (Table 1). The AtheroGene study, involving 1352 patients with SAP and 877 patients with ACS, identified GDF-15 as a new biomarker for risk stratification of patients with SAP and confirmed GDF-15 as a new prognostic biomarker in ACS independent of CV risk factors, number of diseased vessels, renal dysfunction, and other markers (cTnT, NT-proBNP, hs-CRP) [34]. GDF-15 cutoff values, which were used to identify low-risk (<1200 ng/L) or very high-risk (>1800 ng/L) ACS patients, provide information for risk stratification in SAP in this study [34]. Analysis of the ACS study population confirmed that GDF-15 is an independent predictor of CAD mortality, with patients with ACS having significantly higher plasma GDF-15 levels than those with SAP [34]. In the cohort of 3501 patients from the PROVE-IT-TIMI-22 trial, prehospital GDF-15 plasma level was associated with recurrent myocardial infarction and hospitalization for new or worsening heart failure [41] (Table 1). In this regard, GDF-15 did not reflect overlapping disease pathways that might contribute to the development of heart failure after ACS, because prognostic information was independent of clinical predictors and markers like hs-CRP and BNP. As an aside, the PROVE IT-TIMI-22 trial remarkably showed that GDF-15 did not decrease in response to more intensive statin therapy [41].

The Heart and Soul Study with 984 patients examined the effects of psychosocial factors on the health status of patients with stable heart failure (Table 1). In this study, GDF-15 was independently associated with fatal and nonfatal CV events, and hospitalization for heart failure in stable CAD during nearly 9 years of follow-up [39]. In addition, this study demonstrated that higher GDF-15 plasma levels belong to a lower left ventricular ejection fraction (LVEF), diastolic dysfunction, greater inducible ischemia, and lower-body exercise output [39]. The Stabilization of Atherosclerotic Plaque by Initiation of Darapladib Therapy trial (STABILITY) evaluated the effectiveness of the inhibitor of lipoprotein-associated phospholipase A2 (Lp-PLA2), Darapladib, compared with placebo during a median follow-up of 3.7 years, assessing the incidence of CV events in 15,828 patients with stable CAD receiving secondary preventive treatment [37] (Table 1). Additionally, blood samples were obtained from patients with stable CAD, demonstrating that higher GDF-15 plasma concentrations at baseline were associated with an increased event rate of the primary composite end point (death from CVD, nonfatal myocardial infarction, or nonfatal stroke) [37]. In multivariable-adjusted analyses, higher GDF-15 plasma concentrations were associated with age and gender [37]. Risk factors like advanced age, male gender, smoking, hypertension, diabetes mellitus, renal dysfunction, poly-vascular disease, hypertriglyceridemia, leucocytosis, and lower concentrations of hemoglobin and HDL-C were related to GDF-15 plasma concentrations [37]. Similarly, increased GDF-15 plasma concentrations correlated with higher concentrations of NT-proBNP, hs-troponin T, and cystatin C [37, 40]. This study proved that in patients with stable CAD, GDF-15 is an independent risk marker associated with CV and non-CV death [37]. The KAROLA cohort is a prospective study of 1204 CAD patients enrolled in a cardiac rehabilitation program after ACS or coronary artery bypass grafting (CABG) surgery [38] (Table 1). The KAROLA study included patients with stable CAD and a follow-up period of 10 years. This study also demonstrated that baseline GDF-15 levels were associated with the occurrence of a subsequent CV event and all-cause of death after adjustment for established CV risk factors [38].

Data from the above-mentioned clinical trials, indicate that the baseline of GDF-15 plasma concentrations and their changes over 12 months provide important prognostic information for identifying patients at high risk of mortality. In reviewing these various clinical studies, GDF-15 (especially its concentration in plasma/blood) may be suggested as a biomarker for CVD and severity. However, it remains unclear, whether the GDF-15 pathway has therapeutic potential.

## 3. GDF-15, Oxidative Stress and Inflammation

### 3.1 Endothelial Cells

Chronic vascular inflammation, oxidative stress, and endothelial dysfunction are hallmarks of the development and progression of atherosclerotic lesions in coronary arteries resulting in CAD [43, 44, 45]. The imbalance of reactive oxygen species (ROS) and antioxidant defenses is one of the main causes of endothelial dysfunction [43]. Increased NADPH oxidase (Nox) activity uncouples endothelial NO-Synthase (eNOS), increases ROS, and decreases nitric oxide (NO) bioavailability [46]. NO is a strong vasodilator that also inhibits the expression of transcription factors such as NF-κB and adhesion molecules, e.g., intercellular adhesion molecule-1 (ICAM-1) and vascular cell adhesion molecule-1 (VCAM-1) [47]. The relationship between inflammation and oxidative stress in early-stage of human atherosclerosis leads to a cyclic worsening of the condition, as inflammatory processes that attempt to repair oxidative damage increase oxidative stress, which in turn leads to endothelial dysfunction. The cytokine GDF-15 has been shown to enlarge atherosclerotic plaques, increase plaque vulnerability, impair ECs in plaques, and induce endothelial-to-mesenchymal transition (EndMT) [48, 49, 50, 51] (Fig. 1; Table 2, Ref. [52, 53, 54, 55, 56, 57, 58, 59, 60]).

**Table 2.** **Biological function of GDF-15 by different endothelial cell types with clinical relevance**.

Cell Type	Clinical relevance	Effects	Mechanisms/ Molecules	References
HPMEC	PAH	proliferation ↑↓, apoptosis ↓	AKT	[52]
HUVEC	Tumor-angiogenesis	angiogenesis ↑, proliferation ↑, G1-stage cell cycle	phosphorylation of Rb protein, nuclear translocation of E2F-1, AP-1- and E2F-dependent expression of G1 cyclins via PI3K/AKT, JNK, ERK signaling pathways	[53]
HUVEC	Cardiac ischemia	angiogenesis ↑	p53, HIF-1α, VEGF dependent signaling pathway	[59]
HUVEC	diabetes mellitus, hyperglycemia	apoptosis ↓	NF-κB/JNK pathway, PI3K/AKT/eNOS pathway, ROS ↓	[55]
HUVEC	Cardiac disease, cancer	angiogenesis ↓, migration ↓	α${\mathrm{CCN2-mediated angiogenesis,}}_{\text{v}}$${ß}_{3}$ integrins and focal adhesion kinase (FAK)	[58]
HUVEC	regenerative medicine of calvarial defect	proliferation ↑, angiogenesis ↑, oxidative stress	PI3K/AKT, JNK, ERK signaling pathways	[54]
human umbilical vein cell line EA.hy926	Hepatocellular carcinoma	angiogenesis ↑, proliferation ↑, migration ↑, tube formation	Src, AKT, MAPK-, NF-κB-signaling pathway	[60]
HAEC	CVD in women	proliferation ↓	p53 pathway	[56]
Endothelial Colony Forming Cells from adult blood	Senescence	proliferation ↑, migration ↑, oxidative stress	NO ↑, AKT, ERK1/2, SMAD2	[57]

↑- enhancement or promotion; ↓- reduction or inhibition; HPMEC, human pulmonary microvascular endothelial cell; HUVEC, human umbilical veins endothelial cell; HAECs, human aortic endothelial cells.

**Fig. 2.**
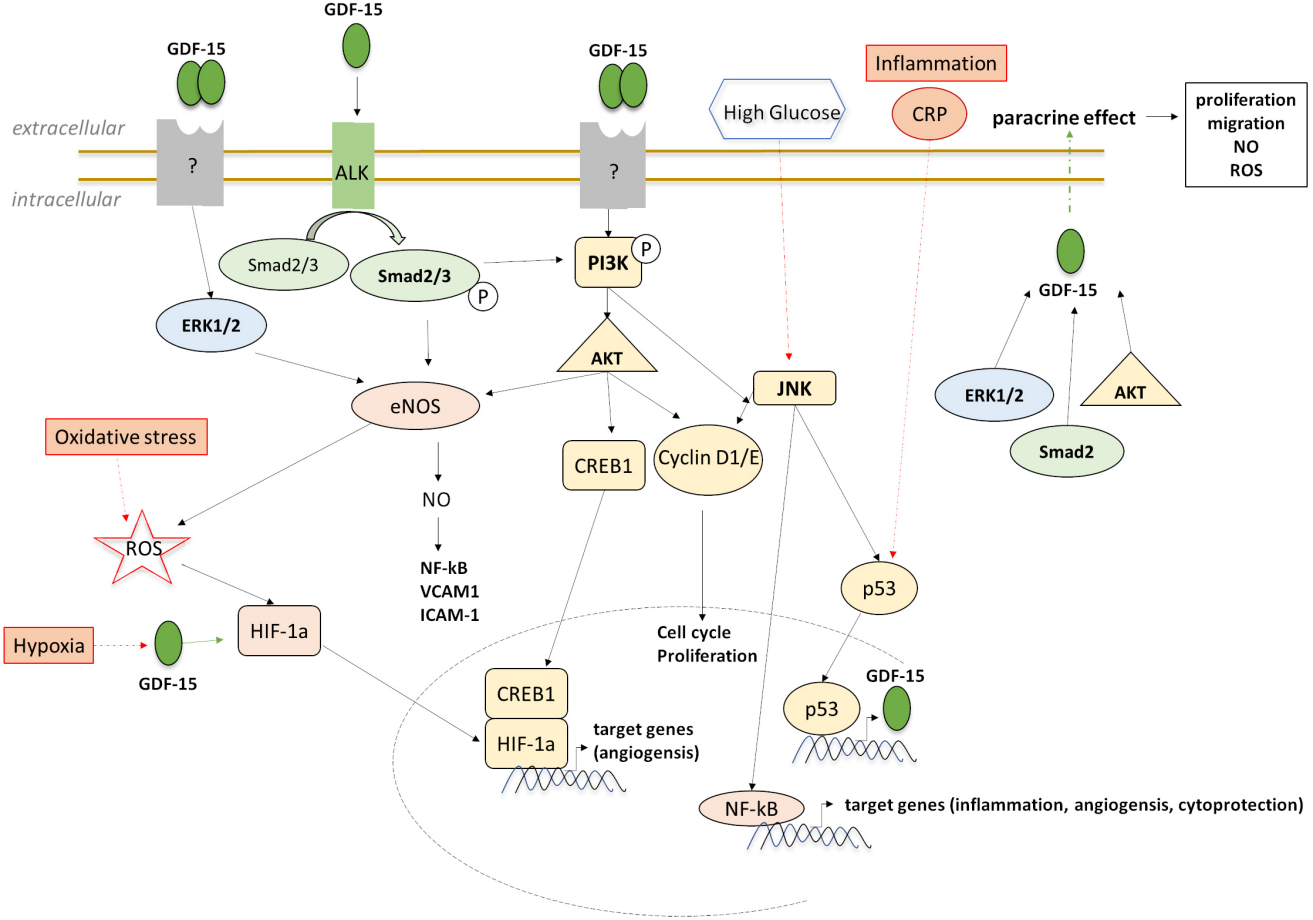
**Downstream targeting and signaling of GDF-15 in ECs in experimental stress-induced models**.

Studies of vascular remodeling in pulmonary arterial hypertension (PAH), which is characterized by endothelial dysfunction with release of vasoactive mediators, growth factors, and cytokines [61], show that GDF-15 is increased in PAH lungs, predominantly located in vascular ECs [52]. PAH is characterized by pulmonary vascular remodeling, progressive arterial stiffening, increased vascular resistance, and right ventricular failure. Animal and human studies suggest with growing evidence that ROS and oxidative stress play a key role in the pathogenesis of PAH [61, 62]. *In vitro* analyses of human pulmonary microvascular endothelial cell (HPMEC) proliferation and apoptosis suggest a role for GDF-15 in endothelial cell homeostasis in PAH patients [52]. HPMEC showed marked upregulation of GDF-15 in hypoxia and laminar shear stress [52]. Recombinant (r) GDF-15 protein decreased apoptotic cell death of HPMEC. In contrast, proliferation was either increased or decreased depending on the concentration of rGDF-15 protein [52] (Table 2). Further studies showed that GDF-15 stimulated the proliferation of human umbilical veins endothelial cells (HUVECs) by upregulating cyclins D1 and E via the phosphoinositide 3-OH kinase (PI3K)/ protein kinase B (AKT) signaling pathway, extracellular signal-regulated kinases (ERK), and c-Jun N-terminal kinase (JNK)-dependent AP-1 and E2F activation signaling pathways [53, 54] (Fig. 2). The effect of GDF-15 against apoptotic cell death might be related to influence on PI3K/AKT/eNOS pathway and NF-κB/JNK pathway. This was demonstrated by *in vitro* studies in HUVECs, where GDF-15 protected against apoptosis, which was induced by high glucose concentration via maintenance of the PI3K/AKT/eNOS pathway and attenuation of the NF-κB/JNK pathway [55]. Clinical studies have shown that GDF-15 plasma levels correlate with the levels of other CV risk biomarkers such as cTnT, NT-proBNP, CRP, possibly indicating a relationship between GDF-15, inflammatory processes, and oxidative stress [30]. Kim *et al*. [56] also demonstrated a molecular relationship between CRP and GDF-15, reporting that GDF-15 expression was increased by CRP via the binding p53 to its promoter region in human aortic endothelial cells (HAECs). Thus, GDF-15 is a direct target gene of p53 through the mediation of CRP [56] (Fig. 2). These data support the predictive role of GDF-15 concerning CVD.

Cell senescence is a mechanism of aging and plays a vital role in the onset and prognosis of CVD [63]. Increasing evidence shows that cell senescence is indispensable in the formation and development of atherosclerosis [63]. To investigate GDF-15 expression, function and role during cellular senescence, Ha *et al*. [57] studied endothelial colony-forming cells (AB-ECFCs) as a model for ECs, because cell senescence is mainly involved in vascular stress and loss of endothelial function. They found that AB-ECFCs expressed higher levels of GDF-15 compared with cord blood colony-forming cells (CB-ECFCs) and that GDF-15 expression progressively increased as AB-ECFCs senescent [57]. Previous studies showed that GDF-15 was overexpressed in radiation-induced senescent HAECs [64]. The paracrine action of GDF-15 promotes AB-ECFC proliferation, migration, and NO production through activation of AKT, ERK, and Mothers against decapentaplegic homolog 2** (**SMAD2) signaling pathways. It induces ROS production independently of nuclear factor-like 2 (NERF2), the major transcription factor regulating antioxidant response [57] (Fig. 2). Ha *et al*. [57] interpreted the paracrine effect of GDF-15 by senescent AB-ECFCs on non-senescent AB-ECFCs as a benefit and claimed that GDF-15 might play a beneficial role in a dysfunctional vasculature by limiting endothelial dysfunction associated with vascular stress.

An increase in endothelial permeability and micro vascularization in the plaque are critical factors in the atherogenesis. Regarding the angiogenic process, Whitson *et al*. [58] described that GDF-15 interacts with connective tissue growth factor 2 (CCN2), inhibits CCN2-mediated angiogenesis, and blocks CCN2-mediated tube formation in HUVECs. However, in hypoxic HUVECs Song *et al*. [59] described that GDF-15 promotes angiogenesis via the hypoxia-inducible factor 1-alpha (HIF-1α)/VEGF-dependent signaling pathway. Furthermore, GDF-15 has been reported to increase the expression of VEGF in a time- and dose-dependent manner, stimulating proliferation and thereby promoting the vascular development of HUVECs [53, 54, 59]. Again, confirming GDF-15 enhances proliferation, migration, and NO production in various endothelial cell types, it also plays an essential role in angiogenesis [53, 54, 59, 60] (Fig. 2).

### 3.2 Leukocytes

An induction of GDF-15 has been reported and described in numerous diseases, such as CVD, cancers, metabolic disorders, rheumatic diseases and viral infection [49, 65, 66, 67, 68]. The majority of these diseases are associated with inflammation and cellular stress.

**Fig. 3.**
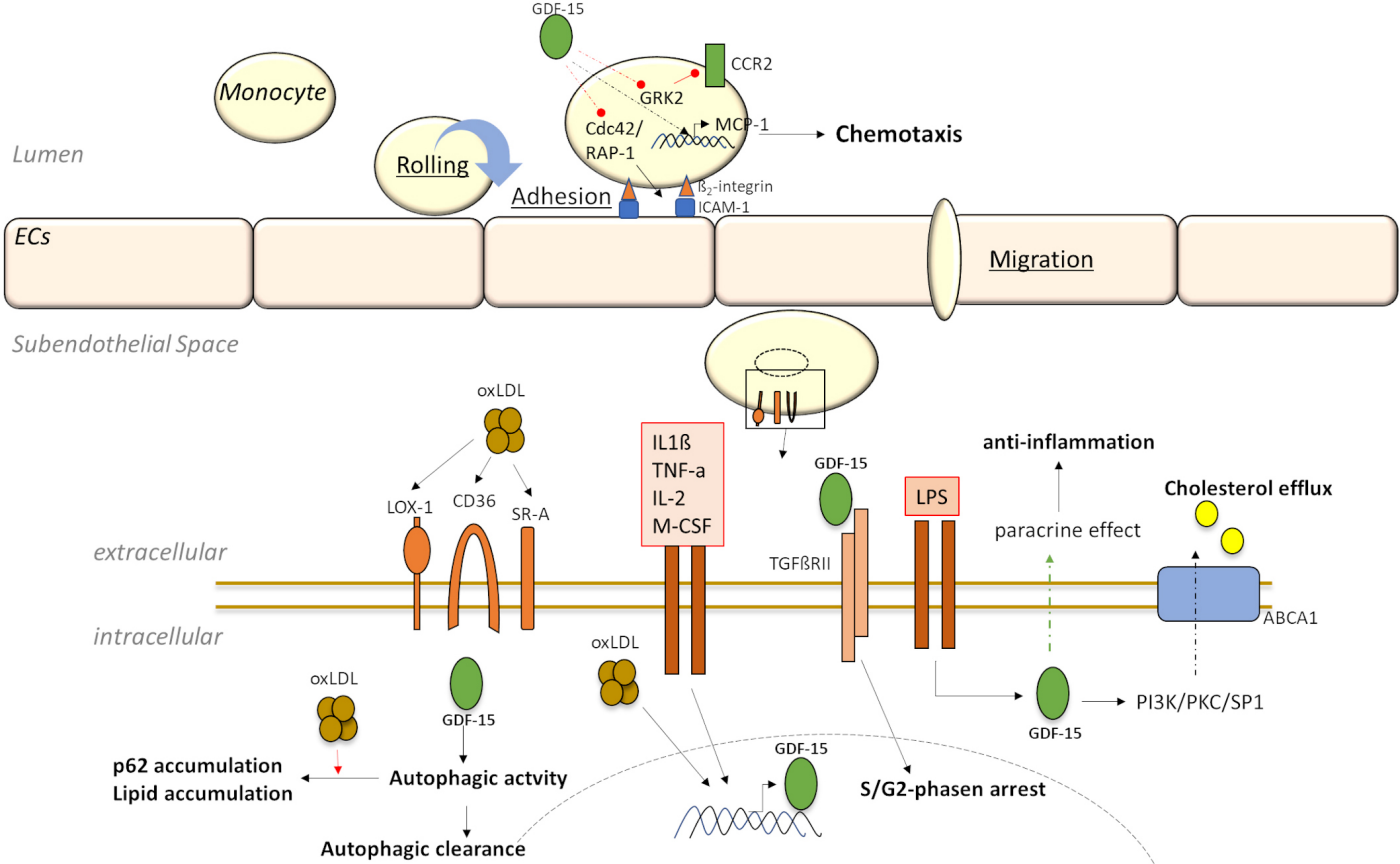
**Downstream targeting and signaling of GDF-15 in monocytes/macrophages in experimental stress-induced models**.

TGF-ß family members, including GDF-15, have effects on cell proliferation, differentiation, apoptosis and inflammation as well as cellular motility and adhesion [69, 70]. The expression of GDF-15 was examined in the human monocytic cell lines U937, KG-1 and THP-1 [1, 71], whereby under (oxidative) stress conditions, such as incubation with trans-retinoic acid (RA) and phorbol 12 myristate 13-acetate (PMA), oxidized low-density lipoprotein (oxLDL), C6-ceramide, or $H_{2}$$O_{2}$ the GDF-15 transcript expression was upregulated in human myelomonocytic cell lines and cultured human macrophages (PBMCs) [1, 13] (Table 3, Ref. [1, 13, 48, 49, 71, 72, 73, 74, 75, 76, 77]). GDF-15, also named NAG-1 and MIC-1, is induced by several anti-inflammatory drugs [78]. Expression of GDF-15 is induced by cytokines involved in macrophages activation like interleukin (IL)-1ß, tumor necrotic factor (TNF)-α, macrophage colony-stimulating factor (M-CSF) and IL-2 (Fig. 3), by nonsteroidal anti-inflammatory drugs (NSAIDs) and by the antidiabetic and anti-inflammatory drug troglitazone [6, 79, 80]. Whereas, interferon (IFN) γ and lipopolysaccharide (LPS) have no effects on GDF-15 expression in U937 and KG-1 [1]. Another study about bacterial and viral infections, as well as sepsis, has described an increased transcription of GDF-15 in an early (1 h–3 h) response to LPS stimulation in ${\mathrm{CD11b}}^{+}$${\mathrm{CD45}}^{+}$ myeloid cells in the liver and bone marrow-derived macrophages from mice [72] (Table 3). Interestingly, the autocrine/paracrine effect of GDF-15 suppresses the LPS-induced TNF-α release in U937 and KG-1 [1]. Therefore, GDF-15 limits LPS-stimulated macrophage activation and inflammation [1] (Fig. 3). Additionally, in serum of LPS-stimulated *hNAG-1/GDF-15* transgenic mice, Kim *et al. * [73] have described a decreased level of pro-inflammatory cytokines. Moreover, GDF-15 reduces the increase of IL‑6, TNF‑α, and IL‑1β expression in serum and liver tissue, and inhibits the activation of the IκBα/NF‑κB pathway by disrupting TGF-β-activated kinase 1 (TAK1) phosphorylation in Kupffer cells [74] (Table 3). Furthermore, GDF-15 prevents LPS/D-galactosamine (D-GalN)-induced cell death, increases inflammatory cell infiltration and serum alanine aminotransferase (ALAT) and aspartate aminotransferase (ASAT) activities in liver tissue [74]. Additionally, GDF-15 has been shown to coordinate tolerance to inflammatory damage through regulating triglyceride metabolism [73]. Kim *et al*. [73] have also described that GDF-15 does not directly inhibit the toll-like receptor (TLR)4/NF-kB pathway in RAW 264.7 and in *${\mathrm{NAG-1}}^{\text{Tg/Lox}}$* peritoneal macrophages, as well as does not affect inflammatory cytokine production from mice Kupffer cells *ex vivo*. But they found that *${\mathrm{NAG-1}}^{\text{Tg/Lox}}$* mice have less white adipose tissue and lower basal leptin level. Leptin is a hormone produced predominantly by adipose cells as a pro-inflammatory cytokine, that appears to be a pivotal mediator of inflammation in mice [81]. Apart from leptin, the adipokine adiponectin was shown to affect atherosclerosis, inflammation and oxidative stress pathways [82]. So, Kim *et al*. [73] have found a link between white adipose tissue mass and inflammation and suggest that GDF-15 may play an anti-inflammatory role in response to LPS stimulation in interaction with leptin (Table 3).

**Table 3.** **Biological function of GDF-15 in different leukocytes, predominantly monocytes / macrophages, with clinical relevance**.

Cell Type	Clinical relevance	Effects	Mechanisms	References
U937, KG-1	paracrine/autocrine effect	Inflammation ↓, Macrophage activation ↓	LPS-induced TNF-α release	[1]
Human peripheral blood mononuclear cells	atherosclerosis	Inflammation, oxidative stress	GDF-15 ↑	[13]
RAW 264.7, bone marrow-derived macrophages	vascular injury	Chemotaxis ↑	S/G2 phase arrest, CCR2	[75]
polymorphonuclear leukocyte	myocardial infarction	leukocyte ${ß}_{2}$ integrin activation ↓, leukocyte arrest ↓, transendothelial migration ↓	GTPase Cdc42, Rap1	[77]
*GDF-15^-/-^*/*ApoE^-/-^* peritoneal macrophages	atherosclerosis	inflammation ↑, apoptosis ↓, atherogenic ↑, Lipid metabolism	IL-6, Caspase-3	[49]
RAW 264.7, *${\mathrm{NAG-1}}^{T\,g/L\,o\,x}$* peritoneal macrophages, Kupffer cells	obesity, intestinal cancer	Leptin expression ↓, inflammation ↓	GDF-15 does not directly inhibit the TLR4/NFκB pathway, TNF-α, IL-6 release	[73]
PMA-differentiated THP-1 macrophages	Foam cells, oxLDL	Cholesterol Efflux ↑	ABCA1, PI3K/PKC/SP1 pathway	[76]
Kupffer cells	acute liver injury	inflammation ↓	IκBα/NF-κB pathway, P-TAK1, IL-6, TNF-α, IL-1β, iNOS	[74]
${\mathrm{CD11b}}^{+}$${\mathrm{CD45}}^{+}$ myeloid cells in the liver, bone marrow-derived macrophages	Viral and bacterial infection, Sepsis	LPS-responds	GDF-15 ↑	[72]
PMA-differentiated THP-1	Foam cells, oxLDL	Autophagy ↑, Lipid accumulation ↑	ATG5, p62-accumulation, LC3II/I	[48, 71]

↑- enhancement or promotion; ↓- reduction or inhibition.

In relation to atherosclerotic research studies, immunohistochemical analyses of human atherosclerotic carotid arteries have demonstrated colocalization of GDF-15 with oxLDL, CD68 immunoreactive cells and apoptosis-relevant proteins [13, 75]. In this context, GDF-15 was up-regulated in murine atherosclerotic lesions during disease progression in a pattern similar to ${\mathrm{CD68}}^{+}$ macrophages [75]. Also, research studies on animals and human clinical trials have shown that oxLDL and induction of apoptosis correlate with an increased GDF-15 protein level and mRNA expression in human macrophages [13, 75] (Fig. 3; Table 3). Therefore, GDF-15 may contribute to oxidative stress-dependent modulation of pro-inflammatory processes in atherosclerotic lesions. Studies with RAW 264.7 macrophages have shown that rGDF-15 promotes the S/G2-phase arrest in a TGF-ßRII-dependent manner (Fig. 3; Table 3) and does not induce apoptosis [75]. Moreover, GDF-15–deficient macrophages appear less prone to oxLDL–induced apoptosis and necrosis [75]. GDF-15 deficiency results in a long-term reduction of atherosclerotic lesions by decreased occurrence of inflammatory ${\mathrm{CD11b}}^{+}$ or ${\mathrm{IL6}}^{+}$ leukocytes as well as an elevated percentage of macrophages, enhanced cell density and decreased p62-accumulation in atherosclerotic lesions of the brachiocephalic trunk in mice [48, 49]. Moreover, it has most recently been shown that, GDF-15 deficiency affects the morphology of atherosclerotic plaques in vessels with deoxygenated blood and low blood pressure, such as the pulmonary trunk (PT), to show a trend decrease of 6.7% in lumen stenoses in the PT of hypercholesterolemic *GDF-15^-/-^*/*ApoE^-/-^* compared with *ApoE^-/-^* mice [50]. Additionally, a significant reduction of the necrotic area in the plaque of GDF-15-deficient mice, with concomitant increases in ${\mathrm{CD68}}^{+}$, α${\mathrm{-actin}}^{+}$, and ${\mathrm{TUNEL}}^{+}$ cells in the plaque of the PT, was demonstrated [50]. Therefore, GDF-15 is thought to be involved in development and progression of atherosclerotic lesions in the brachiocephalic trunk, but also in the PT, likely targeting different mechanisms (e.g., in apoptosis).

In this context,* in vitro* data have proved that GDF-15 deficiency leads to a decreased mRNA expression of apoptosis- or inflammation-relevant cytokines in cultured peritoneal macrophage of mice [49]. Additionally, data from human PMA-differentiated THP-1 macrophages have suggested that GDF-15 is involved in the regulation of lipid homeostasis by regulating autophagic processes [48, 71](Fig. 3; Table 3). Studies using small interfering RNA against GDF-15 (siGDF-15) and recombinant GDF-15 have demonstrated that GDF-15 directly affects autophagic activity in macrophages without affecting lysosomal activity [48, 71]. Also, in combination with oxLDL, GDF-15 affects autophagic processes with consequences for lipid homeostasis in human macrophages [71] (Fig. 3; Table 3), indicating its emerging important pathophysiological role in the development and progression of atherosclerotic plaques. In the context of foam cell formation, another study using THP-1 macrophages has demonstrated that GDF-15 might be a potential target to prevent foam cell formation via the PI3K/PKC/SP1 pathway and promote cholesterol efflux [76] (Fig. 3; Table 3). Therefore, GDF-15 has been shown to regulate apoptosis, autophagy and inflammatory processes of macrophages and is involved in configuring atherosclerotic lesion development.

In terms of the selectin-mediated leukocyte capturing and rolling, followed by the actual transmigration through the endothelium, resulting in chemokine-induced leukocyte arrest [83, 84], GDF-15 is essential to prevent the excessive chemokine-activated leukocyte arrest and transmigration through the endothelium [77]. Additionally, GDF-15 is an inhibitor of leukocyte ${ß}_{2}$-integrin activation via Cdc42 and Rap1 [77] (Fig. 3; Table 3). The interaction of activated β_2_-integrins with ICAM-1 leads to leukocyte arrest on the endothelium and initiates trans-endothelial migration [85]. de Jager *et al*. [75] have concluded that a reduction of macrophage accumulation in plaques of *GDF-15^-/-^* chimeras mice results in an impaired cellular migration and mobility, possibly via C-C chemokine receptor type 2 (CCR2) [75] (Fig. 3; Table 3). CCR2 is a key chemokine receptor for monocyte recruitment at early stage of atherosclerosis and is decreased by GDF-15–deficient macrophages [75]. The direct interaction of GDF-15 with the chemokine receptor CCR2 function suggests that the GDF-15-induced macrophage mobility modulates the CCR2 response [75].

### 3.3 Smooth Muscle Cells

In the context of CAD, smooth muscle cells (SMCs) play a key role in the stability and progression of atherosclerotic plaques. In addition to macrophages and ECs, as cellular sources of GDF-15 production, VSMCs also secrete GDF-15 in response to metabolic and/or oxidative stress or stimulation by pro-inflammatory cytokines [28]. In studies concerning atherosclerotic plaques of the pulmonary trunk of GDF-15 deficient *ApoE^-/-^* mice after 20 weeks cholesterol-enriched diet, Bonaterra *et al*. [50] found an increase in the percentage of α${\mathrm{-actin}}^{+}$ SMCs with a higher percentage of ${\mathrm{CD68}}^{+}$ macrophages and a decreased necrotic core area compared to *ApoE^-/-^* mice.

After a high-fat meal, with elevated postprandial lipemia, a strong upregulation of GDF-15 expression in coronary artery SMCs (CASMCs) by triglyceride-rich lipoproteins (TRL) was observed [86]. The group of TRLs is composed of chylomicrons and very low-density lipoproteins (VLDLs). TRLs and their metabolites are involved in the pathogenesis of atherosclerosis by modulating inflammation, oxidative stress, and foam cell formation [87], as well as inducing cell proliferation [88] and monocyte chemoattractant protein-1 (MCP-1) expression in SMCs [89]. Likewise, TRLs and their metabolites have been detected in atherosclerotic plaques [90].

Hence, more research projects are necessary to understand the direct effects of GDF-15 on VSMCs in the context of atherosclerotic plaque development, progression, and stability.

### 3.4 Cardiomyocytes

Myocardial infarction, a condition associated with CAD, is associated with many deaths [91] and shows upregulation of GDF-15 after acute myocardial infarction [92]. GDF-15 is not constitutively expressed in adult myocardium. Cardiomyocytes produce and secrete GDF-15 only in response to oxidative stress, angiotensin II or inflammatory cytokines, ischemia, and mechanical stretch [28]. Increased plasma levels of GDF-15 can be detected in patients suffering from myocardial infarction, or as a result of injury and heart failure [93, 94]. Among other findings, data from the Women’s Health Study show that serum GDF-15 levels are an independent risk indicator for adverse CV events [93]. In this regard, GDF-15 has cardioprotective effects on cardiomyocytes in ischemic tissue and controls the conversion of cardiac fibroblasts to myofibroblasts during the development of fibrosis [92, 95]. Thus, understanding cellular GDF-15 signaling and crosstalk in cardiac metabolism is a research concern.

**Fig. 4.**
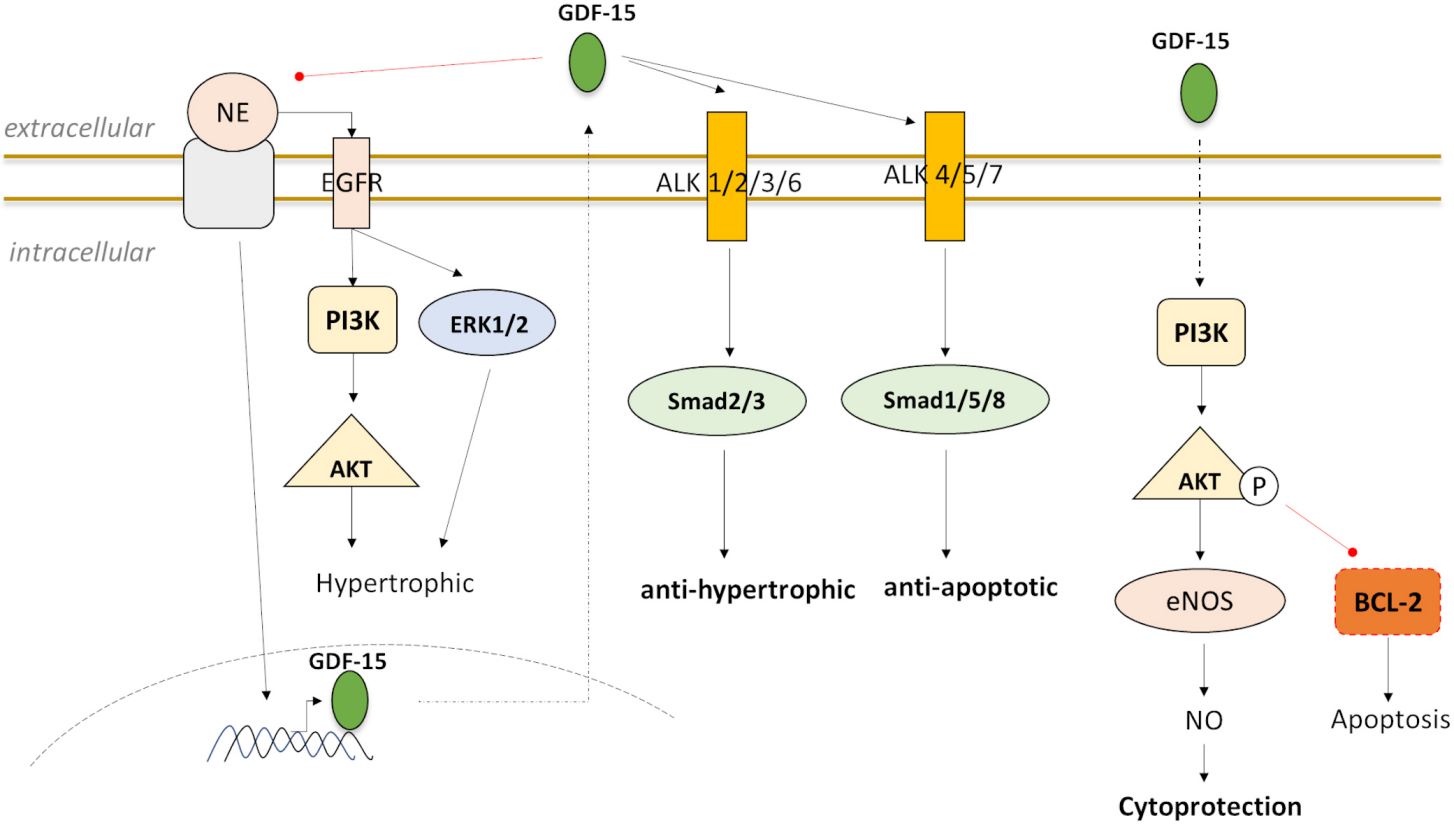
**Downstream targeting and signaling of GDF-15 in experimental stress-induced models that stimulate GDF-15 expression in cardiomyocytes and reveal GDF-15 as a cardioprotective via PI3K-AKT, ERK, and SMAD proteins**.

*In vitro* experiments with immune cells, ECs, and cardiomyocytes from the ventricle suggest that GDF-15 may act as a survival factor on one side and as an inducer of cell death factors on the other [49, 55, 95, 96, 97, 98, 99, 100]. Using GDF-15 gene-targeted mice, endogenous GDF-15 was shown to protect the heart from ischemic/reperfused (I/R) injury [92]. Similarly, cell culture experiments with recombinant GDF-15 showed that cardiomyocytes are protected from hypoxia-induced ischemic injury via PI3K and AKT-dependent signaling pathways [92]. GDF-15 promotes rapid activation by transient Ser473 phosphorylation of AKT in cardiomyocytes, which is accompanied by an increase Ser136 phosphorylation (inactivation) of the AKT downstream target Bcl-2 antagonist of cell death (Bad) [92] (Fig. 4; Table 4, Ref. [92, 95, 99, 101]), a pro-apoptotic protein of the Bcl-2 family [102]. In addition, other PI3K/AKT-independent pathways may be involved in the autocrine/paracrine effects of GDF-15 [95, 99], with GDF-15 transiently activating ERK1/2 in cardiomyocytes [92, 99], but not p38 or JNK [92] (Table 4).

**Table 4.** **Biological function of GDF-15 by cardiomyocytes with clinical relevance**.

Cell Type	Experimental models	process	Effects and mechanisms	References
Ventricular cardiomyocytes from rats	Ischemic injury	Cytoprotective, apoptosis ↓	AKT, PI3K	[92]
Neonatal Ventricular cardiomyocytes from rats	Cardiomyopathy	Cytoprotective, hypertrophic ↓	R-SMAD2, ERK1/2, AKT	[95]
ventricular cardiomyocytes of rat	Heart failure, cardiac remodeling	apoptosis ↓, hypertrophic ↑	PI3K, ERK, and R-SMAD1	[99]
Neonatal rat cardiomyocytes (NRCMs)	Cardiac remodeling	hypertrophic ↓	EGFR, AKT, ERK	[101]

↑- enhancement or promotion; ↓- reduction or inhibition.

Pathological myocardial hypertrophy leads to increased oxygen demand and decreased contractility of the affected ventricle [103]. This usually results in heart failure, as well as an increased risk of myocardial infarction [104, 105]. The hypertrophic signaling effect mediated by GDF-15 via the epidermal growth factor receptor (EGFR), PI3K, AKT, ERK, as well as SMAD proteins is controversial in this regard [95, 99, 101] (Fig. 4; Table 4). Analyses have shown that GDF-15 attenuates norepinephrine (NE)-induced myocardial hypertrophy as well as hypertrophy in cultured rat neonatal ventricular cardiomyocytes through induction of small body size (SMA) and SMAD2/3 phosphorylation and detectable induction of SMAD1/5/8 phosphorylation [95, 101] (Fig. 4; Table 4). NE is known to induce oxidative stress resulting in hypertrophy, apoptosis, and intracellular ${\mathrm{Ca}}^{2+}$ overload in the myocardium [106]. Moreover, *in vivo* and *in vitro* studies show that NE can stimulate the synthesis and release of GDF-15 [101]. Thus, GDF-15 negatively regulates NE-induced myocardial hypertrophy, activation of EGFR, and its signaling pathway [101]. Contrary to the findings of Xu *et al*. [95, 101], GDF-15 triggers hypertrophic growth in rat ventricular cardiomyocytes [99]. Heger *et al*. [99] investigated the different R-SMAD isoforms and found that GDF-15 does not stimulate R-SMAD2 but enhances the phosphorylation of R-SMAD1 (Fig. 4; Table 4). SMAD1 mediates bone morphogenetic protein (BMP) signaling, which is involved in various biological activities including cell growth, apoptosis, development, and immune responses [107]. Furthermore, activation and cardiac-specific overexpression of R-SMAD1 results in smaller myocardial infarct area and reduced apoptotic cell death in cardiomyocytes [108]. Heger *et al*. [99] suggests that GDF-15 gains its anti-apoptotic and pro-hypertrophic character through stimulation of R-SMAD1.

## 4. GDF-15 Receptor

**Fig. 5.**
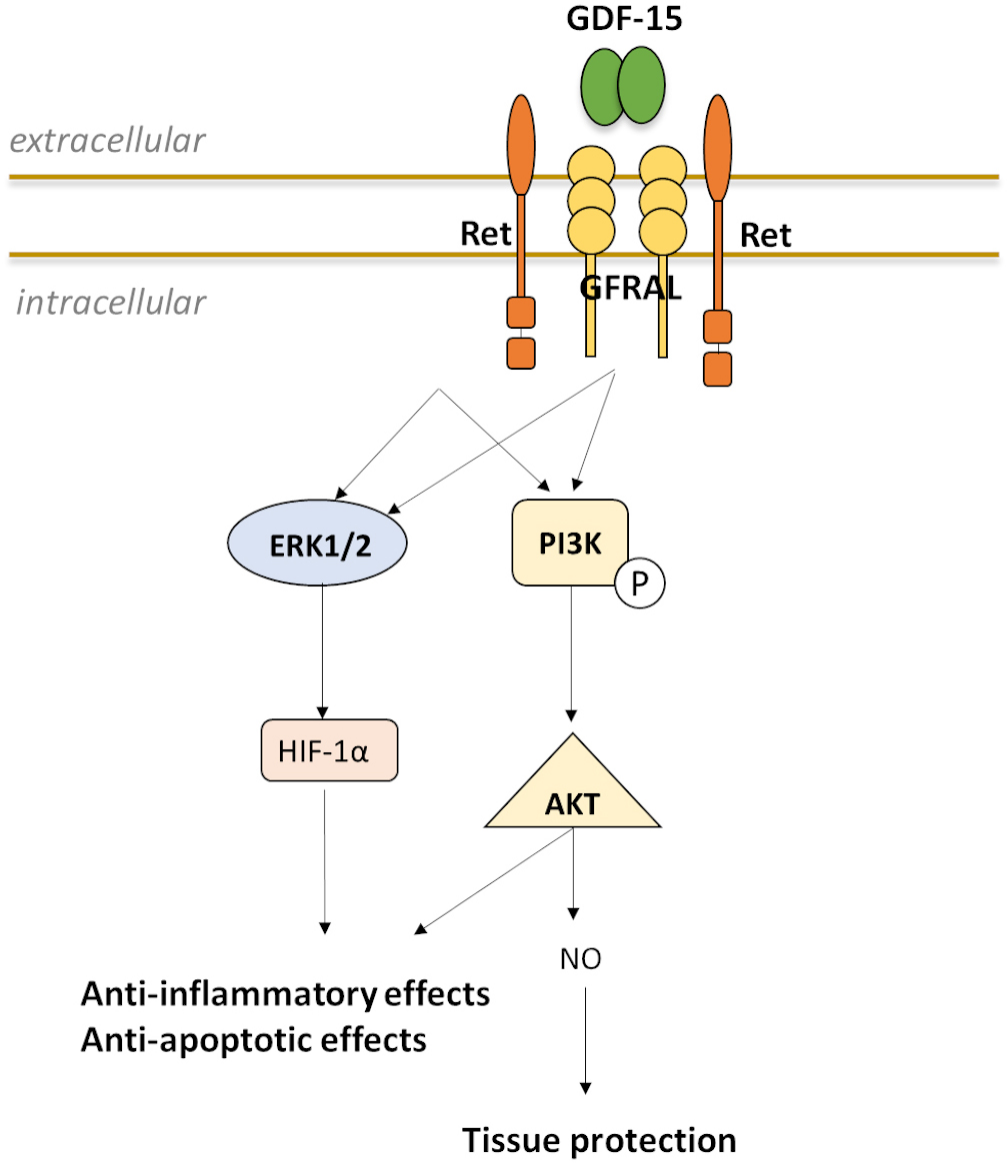
**Possible model for induction and interaction of GDF-15 with GFRAL and the coreceptor RET to enable downstream signal transduction**.

Recently, 5 years ago (in 2017), four research groups simultaneously identified the GDF-15 receptor [109, 110, 111, 112]: By using screening arrays of GDF-15 against glial cell line-derived neurotrophic factor (GDNF) receptors and the orphan GDNF receptors GFRAL (GDNF receptor α-like) and GAS1 (growth-arrest-specific 1) the research groups detected a specific interaction only with GFRAL. GFRAL is a single transmembrane cell surface protein with the highest expression in the brainstem area postrema. It requires interaction with the co-receptor RET, a receptor tyrosine kinase for members of the GDNF receptor family [109, 110, 111, 112]. Mutations of amino acid 87 (valine) or 89 (isoleucine) to arginine leads to loss of binding to GFRAL [110, 111]. There are two isoforms of GFRAL: GFRAL-A and GFRAL-B. GFRAL-A contains a cytoplasmic domain of about 23 amino acids, whose function contributes to stable anchored on the cell membrane [113]. Selective splicing of this cytoplasmic domain produces the truncated protein GFRAL-B [113]. GFRAL-B is secreted into the serum and contains most of the GDF-15 binding structure [114]. Therefore, it is supposed that the soluble GDF-15/GFRAL-B complex could bind to RET located on distant tissues or cells and activate a downstream signaling pathway [114]. To date, it is unknown where and when GFRAL-B might be expressed *in vivo*. However, GDF-15-mediated activation of RET phosphorylation induces signaling through the ERK1/2 and AKT pathways, but not the SMAD pathway [109, 110, 111] (Fig. 5). Considering that GDF-15 is an important regulator of body weight in humans, the research groups found that the metabolic effect depends on the interaction between GDF-15 and GFRAL [109, 110, 111, 112]. In screening experiments of cell lines and human and mouse tissue, as yet, no GFRAL expression has been found outside of the CNS [109, 110, 111, 112, 113]. Only in human tissue low-level expression was identified in testis and adipose tissue [110]. GFRAL was not detected in the aorta, tibia artery or coronary artery. Therefore, to date, it is unclear which cell-specific GDF-15 receptor(s) exist and how they might be involved in atherosclerosis.

## 5. Downstream Signaling of GDF-15 Concerning CAD

The importance and relevance of the GDF-15/TGF-ßRII and the GDF-15/NF-kB pathways in the cardiovascular system are well known [115]. de Jager *et al. * [75] examined the signal transduction cascades for GDF-15 and showed that blockade of TGF-ßRII leads to an abrogation of MCP-1/chemokine (C-C motif) ligand 2 (CCL2) monocyte migration triggered by GDF-15 [75]. This suggests a crucial involvement of GDF-15 in the mechanism of atherosclerosis development and progression. As previously described, the expression of GDF-15 is also upregulated by several pro-inflammatory stimuli in macrophages, including IL-1ß, IL-2, and TNF-α [1]. In a clinical trial, anti-inflammatory therapy with canakinumab targeting the IL-1β-induced innate immunity pathway resulted in a significant reduction of recurrent CV events compared with the placebo group, independent of lowering serum lipid levels [116]. This suggests that an IL-1ß/GDF-15-associated immunity pathway may lead to atherosclerosis and, consequently, CAD. It is therefore speculated that the high plasma GDF-15 levels in CAD patients result from high levels of cytokines such as IL-1ß, TNF-α, and CRP [115]. In turn, inflammatory factors such as IL-1ß or CRP induce GDF-15 expression by regulating p53-binding sites in the GDF-15 promoter and activating downstream NF-KB signaling [56], thereby accelerating the progression of early-stage atherosclerosis and promoting the formation of vulnerable plaques with the possible consequence of CAD.

Recent studies also reveal an essential role of GDF-15 in the mTOR/autophagy pathway in relation to atherosclerotic progression. GDF-15, in combination with oxLDL, impairs autophagic processes with effect on lipid homeostasis in human macrophages [71]. GDF-15 also appears to be an important factor in regulating autophagy in ECs of atherosclerotic lesions [48], with impaired endothelial autophagy in hypercholesterolemic mice abrogating the antiatherogenic effect of blood flow-induced-shear stress, thereby exacerbating the burden of atherogenic plaques and enhancing inflammatory responses [117].

These signaling pathways provide evidence that targeting the pathophysiological activity of GDF-15 may provide novel therapeutic agents for CAD patients. Thus, targeting the GDF-15 pathway is the focus of new therapeutic approaches to combat CAD.

## 6. Conclusions

Early identification of high-risk individuals with CVD is of great importance and could allow timely decisions on preventive measures. Conventional risk factors are enabled for only about half of CAD prevalence. Therefore, it is essential to search for new measurable humoral and genetic markers to improve cardiovascular risk assessment and therapeutic interventions in CAD. GDF-15 is considered one of the most recent promising humoral biomarkers of cardiovascular risk in clinical practice.

Based on the review, from a scientific standpoint, the research perspective is to discover the receptor(s) in the CV system and downstream signaling pathways as the top priority to decipher the activity of GDF-15 in a cell-specific manner. Depending on cell state, cell type, and microenvironment, GDF-15 appears to have both, beneficial and detrimental effects. Clinical studies suggest that patients with elevated GDF-15 levels may benefit from anti-inflammatory, anti-oxidant, or anti-aging therapies. In some studies, increased plasma GDF-15 concentrations over time have already provided strong evidence for poorer prognosis in patients with CAD or heart failure. To date, the reference concentration of GDF-15 in plasma for the healthy general population is not entirely well defined. In this context, further assessment of the effects of environmental and lifestyle factors on GDF-15 concentrations over the life course would provide important insights. Finally, targeted interventions that reduce GDF-15 concentrations could be associated with better health.

## Data Availability

Data and materials are available on request.

## Abbreviations

ACS, acute coronary syndrome; ALAT, alanine aminotransferase; ASAT, aspartate aminotransferase; BAD, Bcl-2 antagonist of cell death; BMP, bone morphogenetic protein; CABG, coronary artery bypass grafting; CAD, coronary artery disease; CASMCs, coronary artery SMCs; CB-ECFCs, cord blood colony-forming cells; CCL2, chemokine (C-C motif) ligand 2; CCN2, connective tissue growth factor 2; CHD, coronary heart disease; CRP, C-reactive protein; cTnT, cardiac troponin-T; CVD, cardiovascular disease; EC, endothelial cell; ECFCs, endothelial colony forming cells; EGFR, epidermal growth factor receptor; EndMT, endothelial-to-mesenchymal transition; ERK, extracellular signal-regulated kinases; GAS1, growth-arrest-specific 1; GDF-15, growth differentiation factor-15; GDNF, glial cell line-derived neurotrophic factor; HAECs, human aortic endothelial cells; HE, Hematoxylin-eosin stain; HIF-1α, hypoxia-inducible factor 1-alpha; HPMEC, human pulmonary microvascular endothelial cell; HUVECs, human umbilical veins; ICAM-1, intercellular adhesion molecule-1; IFN, interferon; IHD, ischemic heart disease; IL, interleukin; I/R, ischemic/reperfused; JNK, c-Jun N-terminal kinase; Lp-PLA2, lipoprotein-associated phospholipase A2; LPS, lipopolysaccharide; LVEF, ventricular ejection fraction; MCP-1, monocyte chemoattractant protein-1; M-CSF, macrophage colony-stimulating factor; MIC, macrophage -inhibitory cytokine-1; NAG-1, nonsteroidal anti-inflammatory drug (NSAID)-activated gene-1; NE, norepinephrine; NERF2, nuclear factor-like 2; NO, nitric oxide; Nox, NADPH oxidase; NSAIDs, nonsteroidal anti-inflammatory drugs; NSTE, non-ST-segment-elevation; NT-proBNP, N-terminal probrain natriuretic peptide; oxLDL, oxidized low-density lipoprotein; PAH, pulmonary arterial hypertension; PDF, prostate-derived factor; PI3K, phosphoinositide 3-OH kinase; PLAB, placental bone morphogenetic protein; PMA, phorbol 12 myristate 13-acetate; PTGF, placental TGF; RA, trans retinoic acid; ROS, reactive oxygen species; SAP, stable angina pectoris; siRNA, small interfering RNA; SMA, small body size; SMAD, Mothers Against Decapentaplegic family; SMCs, smooth muscle cells; STEMI, ST-elevation myocardial infarct; TAK1, TGF-β-activated kinase 1; TLR, toll-like receptor; TNF, tumor necrotic factor; TRL, triglyceride-rich lipoproteins; VCAM-1, vascular cell adhesion molecule-1; VLDLs, very low-density lipoproteins.
